# DEAD-Box RNA Helicase 21 (DDX21) Positively Regulates the Replication of Porcine Reproductive and Respiratory Syndrome Virus via Multiple Mechanisms

**DOI:** 10.3390/v14030467

**Published:** 2022-02-24

**Authors:** Jia Li, Dang Wang, Puxian Fang, Yu Pang, Yanrong Zhou, Liurong Fang, Shaobo Xiao

**Affiliations:** 1 State Key Laboratory of Agricultural Microbiology, College of Veterinary Medicine, Huazhong Agricultural University, Wuhan 430070, China; jialicau@163.com (J.L.); wangdang511@126.com (D.W.); pxfang1990@163.com (P.F.); park_pang@163.com (Y.P.); virus@mail.hzau.edu.cn (Y.Z.); fanglr@mail.hzau.edu.cn (L.F.); 2 The Key Laboratory of Preventive Veterinary Medicine in Hubei Province, Cooperative Innovation Center for Sustainable Pig Production, Wuhan 430070, China

**Keywords:** porcine reproductive and respiratory syndrome virus (PRRSV), DEAD-box helicases 21 (DDX21), interferon, nonstructural protein 1β (nsp1β), interaction, viral replication

## Abstract

The porcine reproductive and respiratory syndrome virus (PRRSV) remains a persistent hazard in the global pig industry. DEAD (Glu-Asp-Ala-Glu) box helicase 21 (DDX21) is a member of the DDX family. In addition to its function of regulating cellular RNA metabolism, DDX21 also regulates innate immunity and is involved in the replication cycle of some viruses. However, the relationship between DDX21 and PRRSV has not yet been explored. Here, we found that a DDX21 overexpression promoted PRRSV replication, whereas knockdown of DDX21 reduced PRRSV proliferation. Mechanistically, DDX21 promoted PRRSV replication independently of its ATPase, RNA helicase, and foldase activities. Furthermore, overexpression of DDX21 stabilized the expressions of PRRSV nsp1α, nsp1β, and nucleocapsid proteins, three known antagonists of interferon β (IFN-β). Knockdown of DDX21 activated the IFN-β signaling pathway in PRRSV-infected cells, suggesting that the effect of DDX21 on PRRSV-encoded IFN-β antagonists may be a driving factor for its contribution to viral proliferation. We also found that PRRSV infection enhanced DDX21 expression and promoted its nucleus-to-cytoplasm translocation. Screening PRRSV-encoded proteins showed that nsp1β interacted with the C-terminus of DDX21 and enhanced the expression of DDX21. Taken together, these findings reveal that DDX21 plays an important role in regulating PRRSV proliferation through multiple mechanisms.

## 1. Introduction

Porcine reproductive and respiratory syndrome virus (PRRSV), which was first identified in the United States in 1987, causes mainly pneumonia and increased mortality in growing pigs, along with reproductive failure in sows (e.g., abortions, weak and stillborn piglets, infertility) [1,2]. PRRSV is a single-stranded positive-sense RNA virus with a lipid spherical envelope, and it belongs to the family *Arteriviridae* [2,3]. The genome of PRRSV is approximately 15 kb in length and encodes at least 10 open reading frames (ORFs) [4,5]. Of these, ORF1a and ORF1b encode two long nonstructural polyproteins, which are subsequently cleaved by viral proteases into 14 nonstructural proteins (nsps): nsp1α, nsp1β, nsp2 to nsp6, nsp7α, nsp7β, and nsp8 to nsp12 [6,7,8]. Other ORFs encode at least eight structural proteins: GP2a, GP3, GP4, GP5, GP5a, envelope (E), matrix (M), and nucleocapsid (N) protein [9,10]. PRRSV is highly sensitive to interferon (IFN) [11]; however, the virus has evolved various strategies to antagonize IFN production and signaling transduction. To date, at least six proteins (nsp1α, nsp1β, nsp2, nsp4, nsp11, and N) encoded by PRRSV have been identified as IFN antagonists; thus, the inhibition of IFN by PRRSV is polygenic [12,13,14,15,16,17].

DEAD (Asp-Glu-Ala-Asp)-box RNA helicases (DDXs) are the largest family of evolutionarily conserved RNA helicases that are involved in a broad array of host processes, including, but not limited to, transcription, pre-mRNA processing, ribosome biogenesis, nuclear mRNA export, translation initiation, and antiviral innate immunity [18,19,20,21,22,23,24]. DDX21, a well-known member of the DDX family, possesses all the signature motifs required for DEAD-helicase function and contains atypical FRGQR repeats in its C-terminus that contribute to its RNA binding and RNA folding activities [25]. Furthermore, growing evidence suggests that, in addition to its function in regulating cellular RNA metabolism, DDX21 also regulates innate immunity and is involved in the replication cycle of some viruses. For example, Hantavirus (HNTV) replication is negatively regulated as DDX21 exerts antiviral effects by enhancing the type I IFN response [26]. Similarly, the replication of dengue virus (DENV) is inhibited during early viral replication when DDX21 travels from the nucleus to the cytoplasm and triggers the activation of IFN-β; however, the DDX21-mediated antiviral effect can be overcome by viral NS2B–NS3 protease complex, which degrades DDX21, thereby promoting DENV replication [27]. Another study recently showed that knockdown of DDX21 negatively regulates IFN-β production, while overexpression of DDX21 did not affect the production of IFN-β, but promotes the replication of vesicular stomatitis virus (VSV); however, infection with VSV caused the caspase-dependent cleavage of DDX21 and promoted its nucleus-to-cytoplasm translocation to negatively regulate the IFN-β signaling pathway by preventing the assembly of the DDX1-DDX21-DHX36 complex [28]. In cells infected with influenza virus (IAV), DDX21 is involved in inhibiting RNA synthesis by binding to the PB1 subunit of viral RNA polymerase and preventing its assembly; however, at later stages of IAV infection, viral NS1 displaces PB1 from DDX21, allowing viral RNA replication to resume [29]. In addition, DDX21 has been demonstrated to be essential for the efficient replication of human immune deficiency virus-1 (HIV-1) by enhancing the binding of viral Rev to RRE [30]. Furthermore, a knockdown of DDX21 significantly inhibited human cytomegalovirus (HCMV) growth and the transcription of viral late genes in human fibroblast cells (MRC5) [31]. As demonstrated by these examples, DDX21 can exhibit either pro- or anti-viral effects depending on the specific virus. However, the relationship between DDX21 and PRRSV has not yet been explored.

In this study, we found that DDX21 positively regulated PRRSV replication. Detailed analyses revealed that the overexpression of DDX21 stabilized the expressions of PRRSV nsp1α, nsp1β, and N protein. Furthermore, a knockdown of DDX21 activated the IFN-β signaling pathway in PRRSV-infected cells. In addition, we also found that PRRSV infection upregulated the expression and promoted the translocation from the nucleus to the cytoplasm of DDX21.

## 2. Materials and Methods

### 2.1. Cells, Virus, and Reagents

HEK-293T cells and MARC-145 cells (kidney cells of the African green monkey) were obtained from the China Center for Type Culture Collection (CCTCC) and cultured at 37 °C in 5% CO_2_ in Dulbecco’s Modified Eagle’s medium (DMEM/High glucose, HyClone, Utah, USA) supplemented with 10% fetal bovine serum (FBS; PAN, USA) and 1% antibiotics (penicillin and streptomycin). iPAM cells [32], an immortalized line of porcine alveolar macrophages, was a kind gift from Xue-Hui Cai. These cells were maintained in RPMI 1640 (Sigma, Saint Quentin-Fallavier, France) supplemented with 10% FBS and 1% antibiotics (penicillin and streptomycin) at 37 °C in a humidified atmosphere of 5% CO_2_. PRRSV strain WUH3 (GenBank accession number HM853673) is a highly pathogenic type 2 PRRSV that was previously isolated from the brains of pigs suffering from hyperthermia syndrome in China at the end of 2006 [33]. Mouse and rabbit monoclonal antibodies against Flag (M185-3L) or HA (M180-3) and rabbit polyclonal antibodies against β-actin (PM053) were purchased from MBL (Beijing, China). Rabbit polyclonal antibody against DDX21 (NB100-1718) was purchased from NOVUS (Shanghai, China). Hsp90α/β Rabbit monoclonal antibodies (A5027) and Lamin A/C Rabbit monoclonal antibodies (A19524) were purchased from ABclone (Wuhan, China). The generations of mouse monoclonal antibodies against PRRSV nsp1β and N protein were described previously [34].

### 2.2. Plasmid Construction

The expression plasmid pCAGGS-Flag-pDDX21 containing the full-length cDNA of porcine DDX21 (pDDX21) was constructed by PCR amplification of the cDNA from PK-15^CD163^ cells, followed by its cloning into pCAGGS with an N-terminal Flag tag (pCAGGS-Flag). The mutants of pDDX21 (pDDX21-K237E, pDDX21-S376L, and pDDX21-M4) were constructed by site-directed mutagenesis and cloned into pCAGGS-Flag to generate the expression plasmids pCAGGS-Flag-pDDX21-K237E, pCAGGS-Flag-pDDX21-S376L, pCAGGS-Flag-pDDX21-M4, respectively. A series of pDDX21 truncation mutants were cloned by overlapping extension PCR using pCAGGS-Flag-pDDX21 as a template. The expression vectors containing the gene encoding the individual PRRSV protein were constructed by cloning each PRRSV gene into a pCAGGS-HA vector as described previously [35].

### 2.3. siRNA and Transfection

The siRNA was designed by Genepharma (Suzhou, China), and the sequences used in our study are listed in Table S1. For transfection, iPAM cells were plated into 24-well plates, and, when 60% confluent, the cells were transfected with 50 pmol of siRNA by using jetPRIME (101000046) (Polyplus, Illkirch, France) in accordance with the manufacturer’s instructions.

### 2.4. RNA Extraction and Quantitative Real-Time PCR (qRT-PCR)

Total cellular RNA was extracted using Trizol reagent (10296010) (Invitrogen, China), after which reverse transcription was performed on 1 µg of RNA from each sample using the Transcription First Strand cDNA synthesis kit (04896866001) (Roche, Mannheim, Germany) to generate cDNA. The resulting cDNA was then used as a template for SYBR green qRT-PCR assays (Applied Biosystems, Foster City, CA, USA). The abundance of individual mRNA transcripts in each sample was assayed three times, and the results were normalized to the internal control. Absolute quantitative mRNA levels of the PRRSV nsp9 gene were calculated by using the amplification curve of its standard plasmid. Relative mRNA expression levels were normalized to the expression of β-actin. All real-time PCR was performed using Power SYBR green PCR master mix (4472908) (Applied Biosystems) in an ABI 7500 real-time PCR system (Applied Biosystems). The qRT-PCR primers used in this study are shown in Table S2.

### 2.5. TCID_50_ Assay

TCID_50_ assays to determine viral titers were performed as described previously [36]. Briefly, MARC-145 cells were seeded in 96-well plates and then infected with serial 10-fold dilutions of supernatants in eight replicates. The plates were incubated for 72–96 h before virus titers were calculated. PRRSV titers are shown as the TCID_50_ per milliliter, calculated using the Reed–Muench method [37].

### 2.6. Indirect Immunofluorescence Assay (IFA)

iPAM cells seeded on microscope coverslips in 24-well plates were co-transfected with pCAGGS-Flag-pDDX21 and the expression plasmids encoding HA-tagged nsp1α, nsp1β, nsp4, nsp12, or N protein. At 30 h post-transfection, the cells were fixed with 4% paraformaldehyde for 10 min at room temperature (RT), permeabilized with methanol for 15 min, blocked in 10% bovine serum albumin for 30 min, and then incubated with mouse monoclonal antibody against HA (M180-3) (dilution 1:400, MBL, Beijing, China) and rabbit polyclonal antibodies against Flag (PM020) (dilution 1:400, MBL, Beijing). After being washed with PBS, the cells were incubated with the secondary antibodies Alexa-fluor-488-conjugated goat anti-mouse IgG (H + L) (A23210) or Alexa-fluor-594-conjugated goat anti-rabbit IgG (H + L) (A23420) (dilution 1:200, Abbkine, Wuhan, China) for 1 h at 37 °C. Finally, nuclei were stained with DAPI. Fluorescent images were acquired with a confocal laser scanning microscope (Fluoviewver.3.1; Olympus, Japan).

### 2.7. Western Blot Analysis

For Western blotting, equivalent amounts of protein samples were subjected to SDS-PAGE and transferred to polyvinylidene difluoride membranes (Millipore, Billerica, MA, USA). These membranes were blocked with 5% nonfat milk for 3 h at RT and then incubated with the indicated primary antibodies. After three washes in TBST, membranes were incubated with horseradish peroxidase (HRP)-conjugated goat anti-rabbit (A0208) or goat anti-mouse antibodies (A0192) (Beyotime, Shanghai) for 1 h at RT. Following washing, proteins were detected using an ECL kit (1705060) (BIO-RAD, USA).

### 2.8. Co-Immunoprecipitation (Co-IP) Assay

At 30 h post-transfection with expression plasmids or at 36 h post-infection (hpi) with PRRSV, cells were collected and lysed by resuspension in 50 mM Tris-HCl, pH 8.0, containing 150 mM NaCl and 1% Triton X-100. For immunoprecipitation, the resulting lysates were rapidly rotated on a rotary shaker for 30 min, and a portion of each supernatant from the lysed cells was used in the whole-cell extract assays. The remaining portions of the supernatants from the lysed cells were immunoprecipitated with the indicated antibodies overnight at 4 °C and then treated with protein A + G agarose beads (P2108) (Beyotime, Shanghai, China) for 4 h at 4 °C. The beads containing the immunoprecipitation samples were washed three times with 1 mL of lysis buffer. Whole-cell extracts and immunoprecipitation samples were resuspended in SDS-PAGE loading buffer, boiled at 95 °C for 5 min, and then subjected to 10% SDS-PAGE and transferred to polyvinylidene difluoride membrane, followed by Western blotting analyses with the indicated antibodies.

### 2.9. Nuclear Cytosol Fractionation Assay

iPAM cells were infected with PRRSV at a multiplicity of infection (MOI) of 0.5. At 36 hpi, the cells were collected, lysed, and fractionated using a Nuclear Cytosol Fractionation kit (P0027) (Beyotime, Shanghai, China), then subjected to a Western blot assay.

### 2.10. Statistical Analysis

GraphPad Prism 8 (GraphPad Software, San Diego, CA, USA) was used for data analysis. Differences between groups were assessed using a two-tailed unpaired *t*-test and were considered statistically significant when the *p*-value was less than 0.05.

## 3. Results

### 3.1. pDDX21 Positively Regulates PRRSV Replication in iPAM Cells

**Figure 1:**
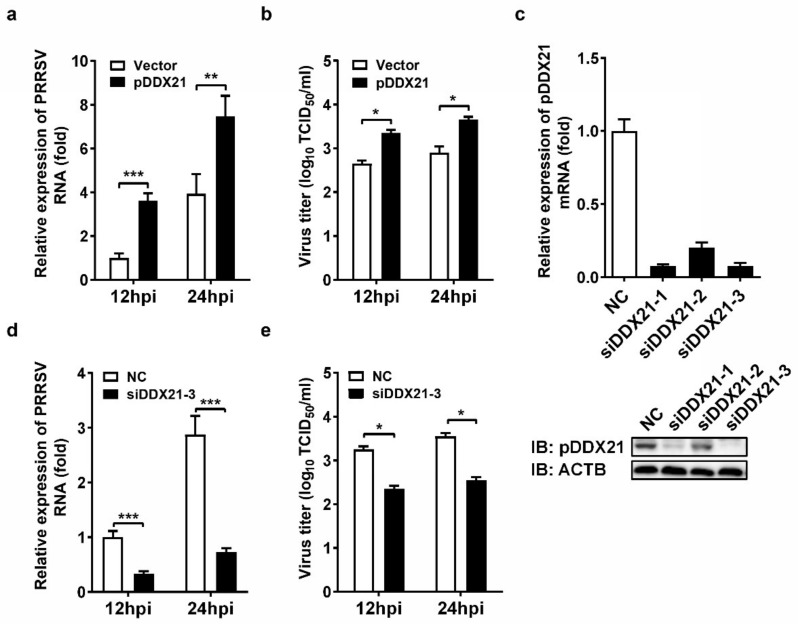
The effect of DDX21 on PRRSV replication. (**a**,**b**) iPAM cells were transfected with a Flag-tagged pDDX21 expression plasmid (3 μg) or empty vectors (3 μg) for 30 h and then infected with PRRSV (MOI = 0.1). At 12 hpi and 24 hpi, the cells were collected for use in qRT-PCR (**a**) and TCID_50_ assay (**b**). (**c**) iPAM cells were transfected with one of three pDDX21-specific siRNAs (siDDX21-1, siDDX21-2, siDDX21-3) or negative control siRNA (NC) for 36 h. The interfering efficiency was measured by qRT-PCR (upper) and Western blotting (lower). (**d**,**e**) iPAM cells were transfected with siDDX21-3 or NC for 24 h and then infected with PRRSV (MOI = 0.1). At 12 hpi and 24 hpi, the cells were collected for use in qRT-PCR (**d**) and TCID_50_ assay (**e**). All experiments were performed in triplicate, and the data are presented as the means ± SD of three independent experiments (* *p* < 0.05; ** *p* < 0.01; *** *p* < 0.001).

To evaluate the role of pDDX21 on PRRSV replication, iPAM cells were transfected with pCAGGS-Flag-pDDX21 for 30 h and then infected with PRRSV (MOI = 0.1). At 12 and 24 hpi, the cells were collected to detect viral genomic RNA by qRT-PCR and determine the viral titer by TCID_50_ assay. The results show that the ectopic expression of pDDX21 significantly increased the numbers of copies of viral genomic RNA (Figure 1a) and viral titers (Figure 1b), suggesting that pDDX21 promotes PRRSV replication. To further confirm the positive effect of pDDX21 on PRRSV replication, we used pDDX21-specific siRNA to knockdown the endogenous pDDX21 expression of iPAM cells. Three siRNAs were designed, and the results of experiments with these siRNAs indicate that siDDX21-3 displayed the highest knockdown efficiency, as evidenced by qRT-PCR and Western blotting data (Figure 1c).

iPAM cells were transfected with siDDX21-3 or negative control siRNA (NC) for 24 h and then infected with PRRSV (MOI = 0.1). At 12 and 24 hpi, the cells were collected for qRT-PCR and TCID_50_ assays. The results show that cells with a knockdown of DDX21 had significantly lower numbers of copies of viral genomic RNA (Figure 1d) and viral titers (Figure 1e) compared with the NC group cells, further demonstrating that pDDX21 positively regulates PRRSV replication in iPAM cells.

### 3.2. pDDX21 Promotes PRRSV Replication Independently of Its ATPase, RNA Helicase, and Foldase Activity

**Figure 2:**
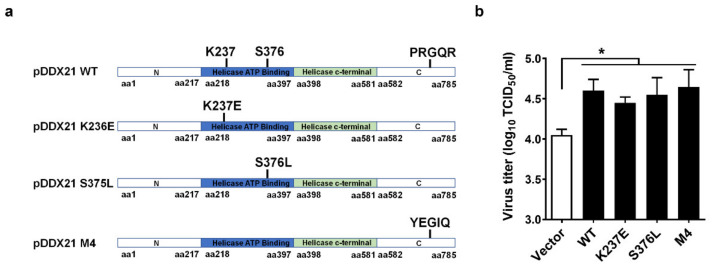
pDDX21 promotes PRRSV replication independently of its ATPase, RNA helicase, and foldase activities. (**a**) Schematic representation of wildtype (WT) pDDX21 and its mutants (K237E, S376L, M4). (**b**) iPAM cells were transfected with pCAGGS-Flag-pDDX21-WT (3 μg), pCAGGS-Flag-pDDX21-K237E (3 μg), pCAGGS-Flag-pDDX21-S376L (3 μg), pCAGGS-Flag-pDDX21-M4 (3 μg), or empty vector (3 μg) for 30 h, and then infected with PRRSV (MOI = 0.1). At 24 hpi, the cells were collected and the viral titers were determined by TCID_50_ assay. Data are presented as the means ± SD of three independent experiments (* *p* < 0.05).

Previous studies reported that DDX21 possesses ATPase activity, RNA helicase activity, and RNA-folding enzyme activity [38,39,40]. To investigate whether the promotion of PRRSV proliferation by pDDX21 depends upon its enzyme activity, we constructed three helicase-dead mutants of pDDX21: pDDX21-K237E (lacking ATPase activity), pDDX21-S376L (lacking RNA helicases activity), and pDDX21-M4 (lacking RNA foldase activity) (Figure 2a). iPAM cells were transfected with expression plasmids encoding wildtype (WT) pDDX21 or its mutants (K237E, S376L, M4) for 30 h and then infected with PRRSV (MOI = 0.1). At 24 hpi, the cells were collected for use in a TCID_50_ assay. The results show that the overexpression of pDDX21 mutants K237E, S376L, and M4 each significantly elevated the viral titers to a similar degree as did the overexpression of WT pDDX21, demonstrating that pDDX21 facilitates PRRSV proliferation independently of its ATPase, RNA helicase, and folding enzyme activities (Figure 2b).

### 3.3. PRRSV Infection Promotes pDDX21 Translocation from the Nucleus to the Cytoplasm

**Figure 3:**
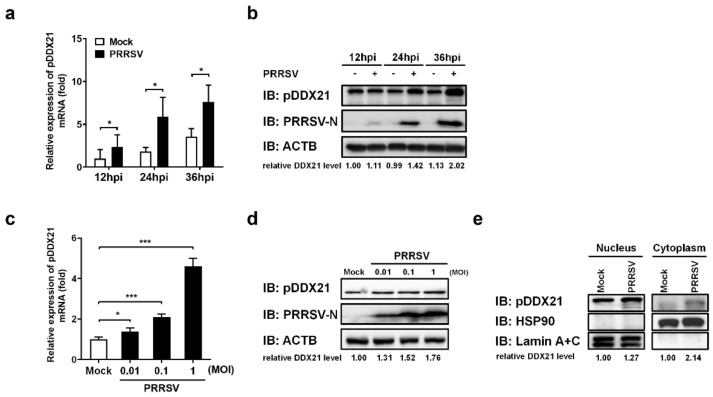
PRRSV infection promotes pDDX21 translocation from the nucleus to the cytoplasm. (**a**,**b**) iPAM cells were infected with PRRSV (MOI = 1). At 12, 24, and 36 hpi, these cells were collected for use in qRT-PCR to determine the number of mRNA copies of pDDX21 (**a**) or in Western blotting with antibodies against DDX21, PRRSV N protein, and β-actin (**b**). (**c**,**d**) iPAM cells were infected with increasing doses of PRRSV (0.01 MOI, 0.1 MOI, or 1.0 MOI). At 24 hpi, these cells were collected for use in qRT-PCR (**c**) and Western blotting (**d**) as described in (**a**,**b**), respectively. (**e**) iPAM cells were grown until they formed a monolayer on 100-mm plates and then infected with PRRSV (MOI = 0.1). These cells were collected at 24 hpi, and a nuclear cytosol fractionation assay was performed to detect the pDDX21 expression in the nucleus or cytoplasm. Data are presented as the means ± SD of three independent experiments (* *p* < 0.05; *** *p* < 0.001). The relative levels of pDDX21 in PRRSV-infected cells as compared with in mock-infected cells were analyzed by ImageJ software, and the ratio is displayed below the images as the fold change.

To investigate whether PRRSV regulates pDDX21 expression, iPAM cells were first infected with PRRSV (MOI = 0.1). Infected cells were collected at 12, 24, and 36 hpi to detect the mRNA and protein expression of pDDX21. The results show that PRRSV infection promoted the transcription (Figure 3a) and protein expression (Figure 3b) of pDDX21 as the infection progressed. We also analyzed the expression of pDDX21 after PRRSV infection with increasing infection doses. As shown in Figure 3c,d, PRRSV infection promotes the transcription and protein expression of pDDX21 in a dose-dependent manner.

Previous studies suggested that changes in subcellular localization are critical for DDX21 to play its role in regulating innate immunity [28,41]. To investigate whether PRRSV infection affects the subcellular localization of pDDX21, iPAM cells were infected with PRRSV for 36 h and then collected for use in a nuclear cytosol fractionation assay, followed by Western blotting analysis. The results show that, compared with the mock-infected group, pDDX21 expression was higher in both the nucleus and the cytoplasm of PRRSV-infected cells (Figure 3e). It should be noted that almost no pDDX21 could be detected in the cytoplasm fraction from mock-infected cells, whereas a clear protein band was observed from PRRSV-infected cells, demonstrating that PRRSV infection promotes the translocation of pDDX21 from the nucleus to the cytoplasm.

### 3.4. pDDX21 Interacts with Multiple PRRSV-Encoded Proteins

**Figure 4:**
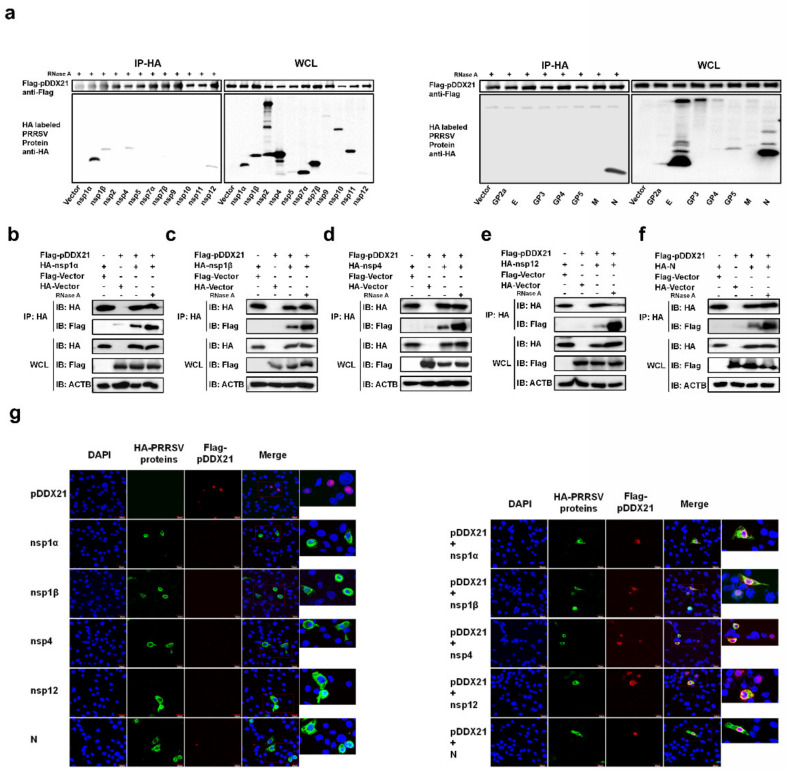
Screening PRRSV proteins for their potential interaction with pDDX21. (**a**) HEK-293T cells were co-transfected with pCAGGS-Flag-pDDX21 (3 μg) and HA-tagged PRRSV protein expression plasmids (3 μg). At 30 h post-transfection, the cells were lysed and immunoprecipitated with an anti-Flag monoclonal antibody. Whole-cell lysate (WCL) and IP complexes were analyzed by immunoblotting with antibodies against Flag, HA, or β-actin. (**b**–**f**) HEK-293T cells were co-transfected with pCAGGS-Flag-pDDX21 (3 μg) and HA-tagged nsp1α (**b**), nsp1β (**c**), nsp4 (**d**), nsp12 (**e**), or N protein (**f**) expression plasmids (3 μg). The cells were lysed at 30 h post-transfection and immunoprecipitated with an anti-HA monoclonal antibody. WCL and IP complexes were analyzed by immunoblotting with antibodies against Flag, HA, or β-actin. (**g**) iPAM cells were co-transfected with pCAGGS-Flag-pDDX21 (1 μg) and HA-tagged nsp1α, 1β, nsp4, nsp12, or N protein expression plasmids (1 μg). At 30 h post-transfection, the cells were fixed for an immunofluorescence assay analysis of pDDX21 and nsp1α, nsp1β, nsp4, nsp12, or N protein using primary antibodies (mouse anti-Flag and rabbit anti-HA), followed by secondary antibodies (AF594-conjugated donkey anti-rabbit and AF488-conjugated donkey anti-mouse). Nuclei were counterstained with DAPI. Fluorescent images were acquired with a confocal laser scanning microscope (Fluoviewver.3.1; Olympus, Japan).

Because pDDX21 was found to promote PRRSV replication and PRRSV infection was also found to enhance pDDX21 expression, it was reasonable to speculate that pDDX21 may achieve this sequence of events through a mechanism of interaction with PRRSV viral protein(s). To confirm this speculation, HEK-293T cells were co-transfected with pCAGGS-Flag-pDDX21 and HA-tagged expression plasmids encoding individual PRRSV proteins, then subjected to a Co-IP assay. Previous studies have demonstrated that DDX21 can bind RNA [42]; thus, to exclude the non-specific interaction mediated by RNA, the cell lysates were treated with RNase A prior to their use in a Co-IP. The results show that pDDX21 interacted with nsp1α, nsp1β, nsp4, nsp12, and N protein (Figure 4a). To further confirm the interactions between pDDX21 and these viral proteins, a reverse Co-IP experiment was also performed. As shown in Figure 4b−f, HA-nsp1α, HA-nsp1β, HA-nsp4, HA-nsp12, and HA-N protein could be efficiently co-immunoprecipitated with Flag-pDDX21 by using an anti-HA antibody.

Although we established that pDDX21 interacts with PRRSV nsp1α, nsp1β, nsp4, nsp12, and N protein, it remained unclear if all these DDX21-interacting proteins affect pDDX21 translocation, as observed in PRRSV-infected cells (Figure 3e). To answer this question, iPAM cells were co-transfected with plasmids expressing Flag-tagged pDDX21 and HA-tagged nsp1α, nsp1β, nsp4, nsp12, or N protein, and then subjected to confocal microscopy. As shown in Figure 4g, when pDDX21 was transfected alone, pDDX21 was localized in the nucleus, while nsp1α, nsp1β, nsp4, nsp12, and N protein were distributed mainly in the cytoplasm. When nsp1α, nsp1β, nsp4, nsp12, or N protein were co-expressed with pDDX21, the subcellular localization of pDDX21 was different: rather than being in the nucleus, pDDX21 now had a cytoplasmic distribution, resulting in the co-localization of pDDX21 with nsp1α, nsp1β, nsp4, nsp12, and N protein (Figure 4g). These results suggest that the nsp1α, nsp1β, nsp4, nsp12, and N protein of PRRSV interact with pDDX21 and redistribute it from the nucleus to the cytoplasm.

### 3.5. PRRSV nsp1β Upregulates DDX21 Transcription and Protein Expression

**Figure 5:**
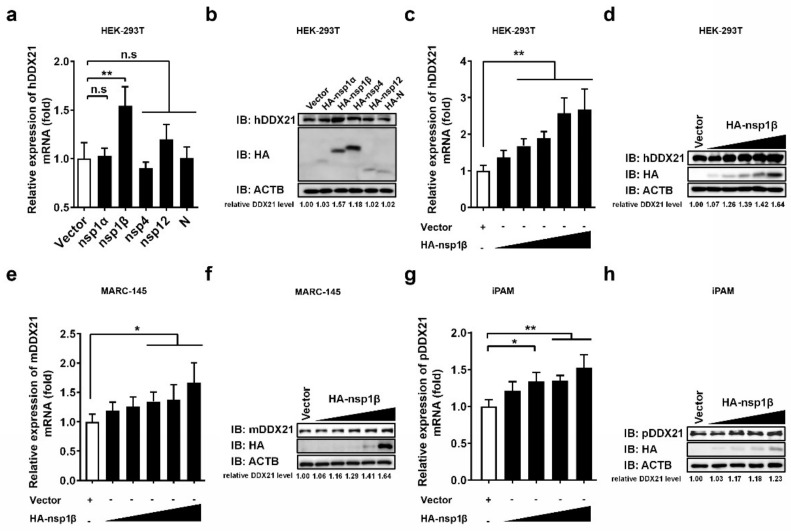
PRRSV nsp1β upregulates DDX21 transcription and expression. (**a**,**b**) HEK-293T cells were transfected with 3 μg of plasmids expressing HA-tagged nsp1α, nsp1β, nsp4, nsp12, or N protein. At 30 h post-transfection, the cells were collected to detect the mRNA expression of DDX21 by qRT-PCR (**a**) or DDX21 protein expression by Western blotting (**b**). (**c**–**h**) HEK-293T cells (**c**,**d**) and MARC-145 cells (**e**,**f**) were transfected with increasing amounts (0.1875 μg, 0.375 μg, 0.75 μg, 1.5 μg, 3 μg) of plasmid encoding HA-tagged nsp1β or empty vector (3 μg). iPAM cells (**g**,**h**) were transfected with increasing amounts (0.375 μg, 0.75 μg, 1.5 μg, 3 μg) of plasmid encoding HA-tagged nsp1β or empty vector (3 μg). At 30 h post-transfection, the cells were collected for use in qRT-PCR (**c**,**e**,**g**) or Western blotting (**d**,**f**,**h**). All experiments were performed in triplicate. The data are shown as the means ± SD of three independent experiments (n.s, no significant differences; * *p* < 0.05; ** *p* < 0.01). The relative levels of DDX21 in experimentally transfected cells as compared with in empty vector-transfected cells were analyzed by ImageJ software, and the resulting ratio is displayed below the images as the fold change.

Because PRRSV infection was found to promote DDX21 expression (Figure 3a–d), and DDX21 was found to interact with PRRSV-encoded nsp1α, 1β, nsp4, nsp12, and N protein (Figure 4a–f), we further investigated whether these DDX21-interacting viral proteins also regulate DDX21 expression. To this end, HEK-293T cells were transfected with plasmids expressing HA-tagged nsp1α, nsp1β, nsp4, nsp12, or N protein, and then subjected to qRT-PCR and Western blot to detect the mRNA and protein expressions, respectively, of DDX21. Among the tested viral proteins, only nsp1β significantly upregulated the DDX21 expression (both mRNA and protein levels; Figure 5a,b, respectively). To further confirm, the upregulation effect of nsp1β on DDX21, HEK-293T cells, MARC-145 cells, and iPAM cells were respectively transfected with increasing doses of nsp1β expression plasmid. The results show that, with increasing nsp1β expression levels, the mRNA and protein levels of DDX21 were significantly upregulated in a dose-dependent manner in all tested cell lines (Figure 5c–h).

### 3.6. nsp1β Interacts with the C-Terminal Domain of pDDX21

**Figure 6:**
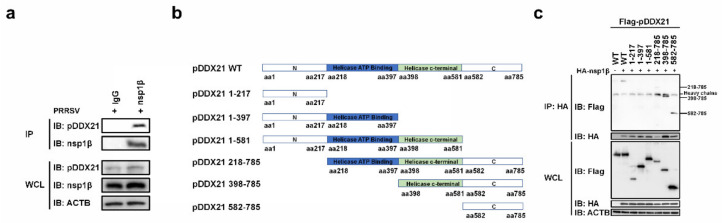
nsp1β interacts with the C-terminal of pDDX21 (**a**) iPAM cells were infected with PRRSV (MOI = 0.5). At 36 hpi, these cells were lysed and immunoprecipitated with anti-nsp1β or anti-IgG monoclonal antibodies. WCL and IP complexes were analyzed by immunoblotting with anti-DDX21, anti-nsp1β, and anti-β-actin antibodies; (**b**) Schematic representation of WT pDDX21 and its truncation mutants. (**c**) HEK-293T cells were co-transfected with expression plasmids of Flag-tagged pDDX21-WT (3 μg) or its truncation mutants (3 μg) and HA-tagged nsp1β (3 μg). At 30 h post-transfection, these cells were lysed and immunoprecipitated with an anti-HA monoclonal antibody. WCL and IP complexes were analyzed by immunoblotting with anti-Flag, anti-HA, or anti-β-actin antibodies.

To investigate whether nsp1β interacts with pDDX21 in the context of PRRSV infection, iPAM cells were infected with PRRSV, and these cells were collected for use in a co-IP assay using a monoclonal antibody against nsp1β. As shown in Figure 6a, the interaction between nsp1β and endogenous pDDX21 could be detected in PRRSV-infected cells.

To further map the domain(s) of pDDX21 interaction with nsp1β, six truncation mutants of pDDX21 were constructed (Figure 6b). HEK-293T cells were co-transfected with HA-tagged nsp1β and Flag-tagged pDDX21 WT or truncation mutants, followed by co-IP experiments. As shown in Figure 6c, the full-length pDDX21, as well as three truncation mutants, pDDX21 218–785 (218 to 785 aa), pDDX21 398–785 (398 to 785 aa), and pDDX21 582–785 (582 to 785 aa), could interact with nsp1β, suggesting that the domain 582–785 aa of pDDX21 is essential for interaction with nsp1β. Taken together, these results illustrate that nsp1β may stabilize the expression of pDDX21 by binding to its C-terminal domain.

### 3.7. pDDX21 Stabilizes the Expression of PRRSV nsp1α, nsp1β, and N Protein

**Figure 7:**
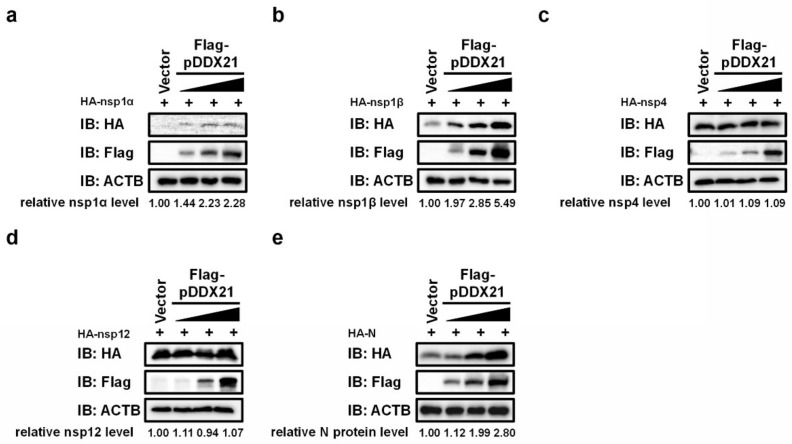
pDDX21 stabilizes the expression of PRRSV nsp1α, nsp1β, and N protein. (**a**–**e**) iPAM cells were co-transfected with increasing concentrations (0.5 µg, 1.0 µg, or 2.0 µg) of Flag-tagged pDDX21 expression plasmid and 1.0 µg of plasmid encoding HA-tagged nsp1α (**a**), nsp1β (**b**), nsp4 (**c**), nsp12 (**d**), or N protein (**e**). At 30 h post-transfection, the cells were collected for use in a Western blotting analysis with antibodies against Flag, HA, or β-actin. The relative levels of nsp1α, nsp1β, nsp4, nsp12, and N protein in transfected cells as compared with the levels in empty vector-transfected cells were analyzed by ImageJ software, and the resulting ratio is displayed below the images as fold change.

To further explore the biological significance of the interaction between pDDX21 and PRRSV-encoded proteins, we examined the effects of pDDX21 on the expressions of nsp1α, nsp1β, nsp4, nsp12, and N protein. iPAM cells were co-transfected with different doses of pCAGGS-Flag-pDDX21 and expression plasmids encoding HA-tagged nsp1α, nsp1β, nsp4, nsp12, or N protein. Cells were harvested at 30 h post-transfection and analyzed by Western blotting. Among the five tested PRRSV proteins, nsp1α, nsp1β, and N protein had expression levels that were significantly upregulated by pDDX21 (Figure 7a,b,e), whereas the expression levels of nsp4 and nsp12 were not significantly altered by pDDX21 (Figure 7c,d).

### 3.8. Knockdown of pDDX21 Activates the IFN-β Signaling Pathway in PRRSV-Infected Cells

**Figure 8:**
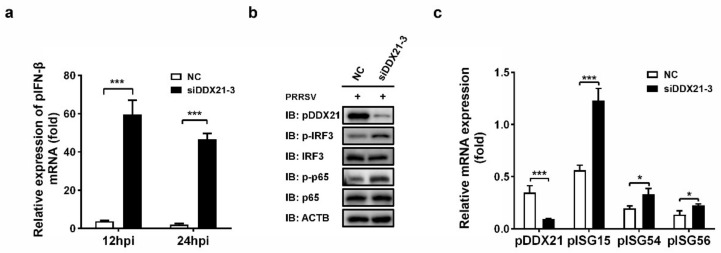
pDDX21 downregulates IFN-β by impairing the activation IRF3 and p65 during PRRSV infection. (**a**) iPAM cells were transfected with siDDX21-3 or NC for 24 h and then infected with PRRSV (MOI = 0.5). At 12 hpi and 24 hpi, the cells were collected to detect pIFN-β mRNA via qRT-PCR. (**b**,**c**) iPAM cells were transfected with siDDX21-3 or NC for 24 h and then infected with PRRSV (MOI = 0.5). At 24 hpi, the cells were collected for use in a Western blotting analysis with the antibodies anti-DDX21, anti-phosphorylated IRF3, anti-total IRF3, anti-phosphorylated p65, anti-total p65, or anti-β-actin (**b**) or in qRT-PCR for detecting the mRNA expression levels of pDDX21, pISG15, pISG54, and pISG56 (**c**). All experiments were performed in triplicate. The data are presented as the means ± SD of three independent experiments (* *p* < 0.05; *** *p* < 0.001).

Because we observed that the overexpression of pDDX21 stabilized the expression of PRRSV nsp1α, nsp1β, and N protein, which are known antagonists of IFN-β [12,13,14,43], we next investigated whether pDDX21 affects porcine IFN-β (pIFN-β) production during PRRSV infection. iPAM cells were transfected with siDDX21-3 or NC for 24 h and then infected with PRRSV (MOI = 0.5). The cells were harvested for detecting the expressions of pIFN-β mRNA and porcine interferon-stimulated genes (pISGs) as well as determining the phosphorylation levels of transcription factor p65 and IRF3. The results show that, after a knockdown of DDX21, the expression levels of pIFN-β, phosphorylated IRF3 (p-IRF3), phosphorylated p65 (p-p65), and several pISGs (pISG15, pISG54, and pISG56) were each significantly increased (Figure 8a–c). Combining these findings with the previous results that nsp1α, nsp1β, and N protein can antagonize pIFN-β production, it is strongly suggested that pDDX21 activates the pIFN-β signaling pathway during PRRSV infection through upregulating the expressions of nsp1α, nsp1β, and N protein. Thus, our data show that pDDX21 is associated with the innate immune responses mediated by PRRSV infection.

## 4. Discussion

PRRSV is a single-stranded, positive-sense RNA virus, and the cellular DEAD-box RNA helicases are involved in all aspects of RNA metabolism. Therefore, it is not surprising that the host DDX proteins may regulate PRRSV viral replication. In previous studies, several DDX proteins have been demonstrated to be associated with PRRSV infection. For example, DDX18 promotes PRRSV replication by interacting with viral nsp2 and nsp10 [44], and DDX5 also positively regulates the replication of PRRSV by interacting with viral nsp9 [45]; furthermore, the overexpression of DDX3X significantly inhibits PRRSV replication [46]. Previous studies also demonstrated that DHX9 is recruited by PRRSV N protein and promotes viral RNA synthesis to facilitate PRRSV proliferation [47]; additionally, a knockdown of DDX19A inhibits PRRSV replication by suppressing NLRP3-dependent inflammasome activation [48]. In the present study, we found that pDDX21 positively regulates PRRSV replication, as demonstrated by the results of pDDX21 overexpression and knockdown of endogenous pDDX21. We also demonstrated that pDDX21 interacts with viral proteins and activates the IFN-β signaling pathway in PRRSV-infected cells, suggesting that pDDX21 regulates PRRSV replication via multiple mechanisms.

Here, we found that pDDX21 interacts with multiple PRRSV-encoded proteins, including nsp1α, nsp1β, nsp4, nsp12, and N protein. To explore the potential significance of these interactions, we examined the effects of pDDX21 on the expression of these five interacting proteins. We initially suspected that these interactions might be associated with RNA because DDX21 is an RNA-binding protein [25,42,49,50,51]. However, the interactions between pDDX21 and nsp1α, nsp1β, nsp4, nsp12, and N protein could still be detected in cells after their treatment with RNase A in co-IP experiments to eliminate RNA contamination, suggesting that these interactions exist even in the absence of RNA. We then found that pDDX21 could stabilize the protein levels of nsp1α, nsp1β, and N protein. Coincidentally, nsp1α, nsp1β, and N protein were previously reported to antagonize type I IFN production. For example, nsp1α inhibits IFN-β expression by degrading CREB-binding protein (CBP) in the nucleus and blocking the recruitment of CBP during assembly of the IFN enhanceosome [51]. A recent study also showed that nsp1α shuttles between the nucleus and cytoplasm and that nuclear export of nsp1α is necessary for its ability to inhibit IFN [52]. In addition to regulating IFN [43], recent reports indicate that nsp1α interacts with swine leukocyte antigen class I (SLA-I) to modulate degradation [53]. Like nsp1α, nsp1β is a nuclear protein that has been shown to inhibit IFN production by suppressing the phosphorylation and nuclear translocation of IRF3 [12]. The latest study found that PRRSV nsp1β prevents the nuclear export of host mRNA into the cytoplasm by disrupting the nuclear pore complex (NPC) of virus-infected cells [52], and further mechanisms revealed that nsp1β interacts with the cellular protein nucleoporin 62 (Nup62) to inhibit host expression of antiviral proteins [54]. PRRSV N protein, like nsp1α and nsp1β, can be distributed in both the nucleus and cytoplasm, and it has been demonstrated to inhibit IFN production by inhibiting the phosphorylation and nuclear translocation of IRF3 [13]. In addition, N protein can also inhibit IFN-β production by suppressing the expression of TRIM25 and the TRIM25-mediated ubiquitination of RIG-I [55].

Previous reports have indicated that DDX21 has diverse biological functions and is involved in almost all processes of RNA metabolism [42]. DDX21 has also been implicated in double-stranded RNA sensing and the associated innate immune response. Additionally, the helicase and RNA chaperon actions of DDX21 in unwinding or unfolding RNA to rearrange the RNA structures for viral genome replication, gene transcription, and translation have been widely reported in the literature, and it is well established that DDX21 is a multifunctional enzyme with RNA-unwinding activity, ATPase activity, RNA foldase activity, R-loop-unwinding activity, and guanine (G)-quadruplex-unwinding activity [25,56,57,58]. For example, during HCMV infection, DDX21 reduces the accumulation of R-loops, which promotes the transcription of HCMV late genes, consequently resulting in the promotion of HCMV growth [31]. DDX21 affects the ribosomal re-initiation of Borna disease virus (BDV) transcripts by interacting with the 5′-untranslated region of X/P mRNA to affect X [59]. Additionally, DDX5 can also utilize its unwinding enzyme activity to help Japanese encephalitis virus (JEV) replication [60]. Therefore, we constructed a series of function-deficient mutants of pDDX21 to determine whether their effects on the virus are related to their enzymatic activities. Unexpectedly, our results showed that the effects of pDDX21 on promoting PRRSV replication are independent of its ATPase, RNA unwinding, and RNA foldase activity.

Another interesting finding from this study is the nucleus-to-cytoplasm translocation of pDDX21 after PRRSV infection. A previous study found that PRRSV infection redistributed DDX18 from the nucleus to the cytoplasm, which may be the primary mechanism for promoting PRRSV replication [44]. DDX21 was first identified as a nucleolar RNA helicase involved in rRNA processing and RNA unwinding [42,58]. However, increasingly more evidence has shown that virus infection or treatment with stimulants, such as poly (I: C) and 3p-hpRNA, promote the translocation of DDX21 from the nucleus to the cytoplasm [28,41,61]. Although the exact mechanism of how pDDX21 translocation influences PRRSV replication is not clear, given that DDX21 possesses G-quadruplex-unwinding activity [25], we speculate that pDDX21 may unwind the viral genome. This possibility needs further investigation.

In summary, we here demonstrate for the first time that, during PRRSV infection, pDDX21 is translocated into the cytoplasm, stabilizes the expressions of PRRSV nsp1α, nsp1β, and N protein, and suppresses the host innate immune response. These results reveal that DDX21 plays an important role in regulating PRRSV replication through multiple mechanisms.

## Data Availability

The datasets generated for this study are available on request to the corresponding author.

## Supplementary Materials

The following supporting information can be downloaded at: https://www.mdpi.com/article/10.3390/v14030467/s1, Table S1: The sequences of siRNA used in this study, Table S2: The primer sequences for qRT-PCR.

## References

[1] Christianson W.T., Collins J.E., Benfield D.A., Harris L., Joo H.S. (1992). Experimental reproduction of swine infertility and respiratory syndrome in pregnant sows. Am. J. Vet. Res..

[2] Kappes M.A., Faaberg K.S. (2015). PRRSV structure, replication and recombination: Origin of phenotype and genotype diversity. Virology.

[3] Wensvoort G., Terpstra C., Pol J.M. (1991). ‘Lelystad agent’—The cause of abortus blauw (mystery swine disease). Tijdschr. Diergeneeskd..

[4] Meulenberg J., Hulst M.M., Meijer E., Moonen P., Besten A.D., Kluyver E., Wensvoort G., Moormann R. (1993). Lelystad virus, the causative agent of porcine epidemic abortion and respiratory syndrome (PEARS), is related to LDV and EAV. Virology.

[5] Wensvoort G., Terpstra C., Pol J.M., ter Laak E.A., Bloemraad M., de Kluyver E.P., Kragten C., van Buiten L., den Besten A., Wagenaar F. (1991). Mystery swine disease in The Netherlands: The isolation of Lelystad virus. Vet. Q..

[6] Fang Y., Snijder E.J. (2010). The PRRSV replicase: Exploring the multifunctionality of an intriguing set of nonstructural proteins. Virus Res..

[7] Li Y., Tas A., Snijder E.J., Fang Y. (2012). Identification of porcine reproductive and respiratory syndrome virus ORF1a-encoded non-structural proteins in virus-infected cells. J. Gen. Virol..

[8] Li Y., Tas A., Sun Z., Snijder E.J., Fang Y. (2015). Proteolytic processing of the porcine reproductive and respiratory syndrome virus replicase. Virus Res..

[9] Snijder E.J., Kikkert M. (2008). Arteriviruses. Encyclopedia of Virology.

[10] Firth J. (2011). Rheumatoid arthritis: Diagnosis and multidisciplinary management. Br. J. Nurs..

[11] Overend C., Mitchell R., He D., Rompato G., Grubman M.J., Garmendia A.E. (2007). Recombinant swine beta interferon protects swine alveolar macrophages and MARC-145 cells from infection with Porcine reproductive and respiratory syndrome virus. J. Virol..

[12] Beura L.K., Sarkar S.N., Kwon B., Subramaniam S., Jones C., Pattnaik A.K., Osorio F.A. (2010). Porcine reproductive and respiratory syndrome virus nonstructural protein 1beta modulates host innate immune response by antagonizing IRF3 activation. J. Virol..

[13] Sagong M., Lee C. (2011). Porcine reproductive and respiratory syndrome virus nucleocapsid protein modulates interferon-β production by inhibiting IRF3 activation in immortalized porcine alveolar macrophages. Arch. Virol..

[14] Wang R., Nan Y., Yu Y., Zhang Y.J. (2013). Porcine reproductive and respiratory syndrome virus Nsp1β inhibits interferon-activated JAK/STAT signal transduction by inducing karyopherin-α1 degradation. J. Virol..

[15] Li H., Zheng Z., Zhou P., Zhang B., Shi Z., Hu Q., Wang H. (2010). The cysteine protease domain of porcine reproductive and respiratory syndrome virus non-structural protein 2 antagonizes interferon regulatory factor 3 activation. J. Gen. Virol..

[16] Chen J., Wang D., Sun Z., Gao L., Zhu X., Guo J., Xu S., Fang L., Li K., Xiao S. (2019). Arterivirus nsp4 Antagonizes Interferon Beta Production by Proteolytically Cleaving NEMO at Multiple Sites. J. Virol..

[17] Wang D., Chen J., Yu C., Zhu X., Xu S., Fang L., Xiao S. (2019). Porcine Reproductive and Respiratory Syndrome Virus nsp11 Antagonizes Type I Interferon Signaling by Targeting IRF9. J. Virol..

[18] Linder P., Jankowsky E. (2011). From unwinding to clamping—The DEAD box RNA helicase family. Nat. Rev. Mol. Cell Biol..

[19] Diot C., Fournier G., Dos Santos M., Magnus J., Komarova A., van der Werf S., Munier S., Naffakh N. (2016). Influenza A Virus Polymerase Recruits the RNA Helicase DDX19 to Promote the Nuclear Export of Viral mRNAs. Sci. Rep..

[20] Ullah R., Li J., Fang P., Shaobo X., Fang L. (2021). DEAD/H-box helicases: Anti-viral and pro-viral roles during infections. Virus Res..

[21] Meier-Stephenson V., Mrozowich T., Pham M., Patel T.R. (2018). DEAD-box helicases: The Yin and Yang roles in viral infections. Biotechnol. Genet. Eng. Rev..

[22] Taschuk F., Cherry S. (2020). DEAD-Box Helicases: Sensors, Regulators, and Effectors for Antiviral Defense. Viruses.

[23] Dehghani M., Lasko P. (2017). Multiple Functions of the DEAD-Box Helicase Vasa in Drosophila Oogenesis. Results Probl. Cell Differ..

[24] Ali M.A.M. (2021). The DEAD-box protein family of RNA helicases: Sentinels for a myriad of cellular functions with emerging roles in tumorigenesis. Int. J. Clin. Oncol..

[25] McRae E.K.S., Booy E.P., Moya-Torres A., Ezzati P., Stetefeld J., McKenna S.A. (2017). Human DDX21 binds and unwinds RNA guanine quadruplexes. Nucleic Acids Res..

[26] Ma H.W., Ye W., Chen H.S., Nie T.J., Cheng L.F., Zhang L., Han P.J., Wu X.A., Xu Z.K., Lei Y.F. (2017). In-Cell Western Assays to Evaluate Hantaan Virus Replication as a Novel Approach to Screen Antiviral Molecules and Detect Neutralizing Antibody Titers. Front. Cell Infect. Microbiol..

[27] Dong Y., Ye W., Yang J., Han P., Wang Y., Ye C., Weng D., Zhang F., Xu Z., Lei Y. (2016). DDX21 translocates from nucleus to cytoplasm and stimulates the innate immune response due to dengue virus infection. Biochem. Biophys. Res. Commun..

[28] Wu W., Qu Y., Yu S., Wang S., Yin Y., Liu Q., Meng C., Liao Y., Ur Rehman Z., Tan L. (2021). Caspase-Dependent Cleavage of DDX21 Suppresses Host Innate Immunity. mBio.

[29] Chen G., Liu C.H., Zhou L., Krug R.M. (2014). Cellular DDX21 RNA helicase inhibits influenza A virus replication but is counteracted by the viral NS1 protein. Cell Host Microbe..

[30] Naji S., Ambrus G., Cimermančič P., Reyes J.R., Johnson J.R., Filbrandt R., Huber M.D., Vesely P., Krogan N.J., Yates J.R. (2012). Host cell interactome of HIV-1 Rev includes RNA helicases involved in multiple facets of virus production. Mol. Cell Proteom..

[31] Hao H., Han T., Xuan B., Sun Y., Tang S., Yue N., Qian Z. (2019). Dissecting the Role of DDX21 in Regulating Human Cytomegalovirus Replication. J. Virol..

[32] Wang T.Y., Liu Y.G., Li L., Wang G., Wang H.M., Zhang H.L., Zhao S.F., Gao J.C., An T.Q., Tian Z.J. (2018). Porcine alveolar macrophage CD163 abundance is a pivotal switch for porcine reproductive and respiratory syndrome virus infection. Oncotarget.

[33] Li B., Fang L., Liu S., Zhao F., Jiang Y., He K., Chen H., Xiao S. (2010). The genomic diversity of Chinese porcine reproductive and respiratory syndrome virus isolates from 1996 to 2009. Vet. Microbiol..

[34] Song T., Fang L., Wang D., Zhang R., Zeng S., An K., Chen H., Xiao S. (2016). Quantitative interactome reveals that porcine reproductive and respiratory syndrome virus nonstructural protein 2 forms a complex with viral nucleocapsid protein and cellular vimentin. J. Proteom..

[35] Ke W., Fang L., Tao R., Li Y., Jing H., Wang D., Xiao S. (2019). Porcine Reproductive and Respiratory Syndrome Virus E Protein Degrades Porcine Cholesterol 25-Hydroxylase via the Ubiquitin-Proteasome Pathway. J. Virol..

[36] Ke W., Fang L., Jing H., Tao R., Wang T., Li Y., Long S., Wang D., Xiao S. (2017). Cholesterol 25-Hydroxylase Inhibits Porcine Reproductive and Respiratory Syndrome Virus Replication through Enzyme Activity-Dependent and -Independent Mechanisms. J. Virol..

[37] Pizzi M. (1950). Sampling variation of the fifty percent end-point, determined by the Reed-Muench (Behrens) method. Hum. Biol..

[38] Valdez B.C. (2000). Structural domains involved in the RNA folding activity of RNA helicase II/Gu protein. Eur. J. Biochem..

[39] Valdez B.C., Henning D., Perumal K., Busch H. (1997). RNA-unwinding and RNA-folding activities of RNA helicase II/Gu—Two activities in separate domains of the same protein. Eur. J. Biochem..

[40] Ou Y., Fritzler M.J., Valdez B.C., Rattner J.B. (1999). Mapping and characterization of the functional domains of the nucleolar protein RNA helicase II/Gu. Exp. Cell Res..

[41] Zhang Z., Kim T., Bao M., Facchinetti V., Jung S.Y., Ghaffari A.A., Qin J., Cheng G., Liu Y.J. (2011). DDX1, DDX21, and DHX36 helicases form a complex with the adaptor molecule TRIF to sense dsRNA in dendritic cells. Immunity.

[42] Calo E., Flynn R.A., Martin L., Spitale R.C., Chang H.Y., Wysocka J. (2015). RNA helicase DDX21 coordinates transcription and ribosomal RNA processing. Nature.

[43] Chen Z., Liu S., Sun W., Chen L., Yoo D., Li F., Ren S., Guo L., Cong X., Li J. (2016). Nuclear export signal of PRRSV NSP1α is necessary for type I IFN inhibition. Virology.

[44] Jin H., Zhou L., Ge X., Zhang H., Zhang R., Wang C., Wang L., Zhang Z., Yang H., Guo X. (2017). Cellular DEAD-box RNA helicase 18 (DDX18) Promotes the PRRSV Replication via Interaction with Virus nsp2 and nsp10. Virus Res..

[45] Zhao S., Ge X., Wang X., Liu A., Guo X., Zhou L., Yu K., Yang H. (2015). The DEAD-box RNA helicase 5 positively regulates the replication of porcine reproductive and respiratory syndrome virus by interacting with viral Nsp9 in vitro. Virus Res..

[46] Chen Q., Liu Q., Liu D., Wang D., Chen H., Xiao S., Fang L. (2014). Molecular cloning, functional characterization and antiviral activity of porcine DDX3X. Biochem. Biophys. Res. Commun..

[47] Liu L., Tian J., Nan H., Tian M., Li Y., Xu X., Huang B., Zhou E., Hiscox J.A., Chen H. (2016). Porcine Reproductive and Respiratory Syndrome Virus Nucleocapsid Protein Interacts with Nsp9 and Cellular DHX9 To Regulate Viral RNA Synthesis. J. Virol..

[48] Li J., Hu L., Liu Y., Huang L., Mu Y., Cai X., Weng C. (2015). DDX19A Senses Viral RNA and Mediates NLRP3-Dependent Inflammasome Activation. J. Immunol..

[49] Holmström T.H., Mialon A., Kallio M., Nymalm Y., Mannermaa L., Holm T., Johansson H., Black E., Gillespie D., Salminen T.A. (2008). c-Jun Supports Ribosomal RNA Processing and Nucleolar Localization of RNA Helicase DDX21. J. Biol. Chem..

[50] Henning D., So R.B., Jin R., Lau L.F., Valdez B.C. (2003). Silencing of RNA Helicase II/Guα Inhibits Mammalian Ribosomal RNA Production. J. Biol. Chem..

[51] Han M., Du Y., Song C., Yoo D. (2013). Degradation of CREB-binding protein and modulation of type I interferon induction by the zinc finger motif of the porcine reproductive and respiratory syndrome virus nsp1α subunit. Virus Res..

[52] Han M., Ke H., Zhang Q., Yoo D. (2017). Nuclear imprisonment of host cellular mRNA by nsp1β protein of porcine reproductive and respiratory syndrome virus. Virology.

[53] Du J., Ge X., Liu Y., Jiang P., Wang Z., Zhang R., Zhou L., Guo X., Han J., Yang H. (2016). Targeting Swine Leukocyte Antigen Class I Molecules for Proteasomal Degradation by the nsp1α Replicase Protein of the Chinese Highly Pathogenic Porcine Reproductive and Respiratory Syndrome Virus Strain JXwn06. J. Virol..

[54] Ke H., Han M., Kim J., Gustin K.E., Yoo D. (2019). Porcine Reproductive and Respiratory Syndrome Virus Nonstructural Protein 1 Beta Interacts with Nucleoporin 62 To Promote Viral Replication and Immune Evasion. J. Virol..

[55] Zhao K., Li L.-W., Jiang Y.-F., Gao F., Zhang Y.-J., Zhao W.-Y., Li G.-X., Yu L.-X., Zhou Y.-J., Tong G.-Z. (2019). Nucleocapsid protein of porcine reproductive and respiratory syndrome virus antagonizes the antiviral activity of TRIM25 by interfering with TRIM25-mediated RIG-I ubiquitination. Vet. Microbiol..

[56] Flores-Rozas H., Hurwitz J. (1993). Characterization of a new RNA helicase from nuclear extracts of HeLa cells which translocates in the 5′ to 3′ direction. J. Biol. Chem..

[57] Valdez B.C., Henning D., Busch R.K., Woods K., Flores-Rozas H., Hurwitz J., Perlaky L., Busch H. (1996). A nucleolar RNA helicase recognized by autoimmune antibodies from a patient with watermelon stomach disease. Nucleic Acids Res..

[58] Song C., Hotz-Wagenblatt A., Voit R., Grummt I. (2017). SIRT7 and the DEAD-box helicase DDX21 cooperate to resolve genomic R loops and safeguard genome stability. Genes Dev..

[59] Watanabe Y., Ohtaki N., Hayashi Y., Ikuta K., Tomonaga K. (2009). Autogenous translational regulation of the Borna disease virus negative control factor X from polycistronic mRNA using host RNA helicases. PLoS Pathog..

[60] Li C., Ge L.-L., Li P.-P., Wang Y., Sun M.-X., Huang L., Ishag H., Di D.-D., Shen Z.-Q., Fan W.X. (2013). The DEAD-box RNA helicase DDX5 acts as a positive regulator of Japanese encephalitis virus replication by binding to viral 3′ UTR–ScienceDirect. Antivir. Res..

[61] Abdullah S.W., Wu J., Zhang Y., Bai M., Guan J., Liu X., Sun S., Guo H. (2021). DDX21, a Host Restriction Factor of FMDV IRES-Dependent Translation and Replication. Viruses.

