# Supplementary material for: DEAD-Box RNA Helicase 21 (DDX21) Positively Regulates the Replication of Porcine Reproductive and Respiratory Syndrome Virus via Multiple Mechanisms
Source: Viruses. 2022 Feb 24;14(3):467. doi: 10.3390/v14030467 (PMC8949431; doi:10.3390/v14030467)
Supplement: Supplementary file 1 [file viruses-14-00467-s001.zip › viruses-1615404-supplementary.pdf]

Supplementary Materials

Table S1. The sequences of siRNA used in this study.

Gene name	siRNA sequence (sense) (5′–3′)	siRNA sequence (anti-sense) (5′–3′)
siDDX21-1	CCCUUUGAUUGAGAAACUUTT	AAGUUUCUCAAUCAAAGGGTT
siDDX21-2	GGACACCAGGUCGUAUUAATT	UUAAUACGACCUGGUGUCCTT
siDDX21-3	GCAACUGGGUGAGGAUAUUTT	AAUAUCCUCACCCAGUUGCTT
siNegative control	ACGUGACACGUUCGGAGAATT	UUCUCCGAACGUGUCACGUTT

Table S2. The primer sequences for qRT-PCR.

Gene name	Forward primer sequence (5′–3′)	Reverse primer sequence (5′–3′)
PRRSV-nsp9	ACCCTAGGACCTGTGAAC	GGCGAGTAACTTAGGAGATG
pDDX21	CTTCTCAAAGCCCGTGGAGT	GTCTTCCCAGTTCCTGTCCG
pGAPDH	CTTCCTGGGCATGGAGTCC	GGCGCGATGATCTTGATCTTC
hDDX21	AAACTCCGTAGTGACGCTGG	TCAACTTCAGAAGGCTCTGCTT
hGAPDH	TCATGACCACAGTCCATGC	GGATGACCTTGCCCACAGCC
mDDX21	TCATCAAGGACGCACTATCATCT	CCTTTCAGGGTGATTTCCCTTT
mGAPDH	TCATGACCACAGTCCATGCC	GGATGACCTTGCCCACAGCC
